# Prognostic Factors and Nomograms for Overall and Cancer-Specific Survival of Patients with Uveal Melanoma without Metastases: A SEER Analysis of 4119 Cases

**DOI:** 10.1155/2022/1874336

**Published:** 2022-09-14

**Authors:** Xin Liu, Chang Liu, Yue Shang, Lin Yang, Fengling Tan, Yong Lv

**Affiliations:** ^1^Department of Ophthalmology, Children's Hospital Affiliated to Zhengzhou University, Henan Children's Hospital, Zhengzhou Children's Hospital, Zhengzhou 450018, China; ^2^Eye Hospital of Shandong First Medical University, Shandong Eye Institute, Shandong First Medical University & Shandong Academy of Medical Sciences, Jinan 250021, China; ^3^Department of Ophthalmology, First Affiliated Hospital of Zhengzhou University, Zhengzhou 450052, China

## Abstract

**Purpose:**

To determine prognostic factors for patients with uveal melanoma without metastases and to construct nomograms to predict their 3- and 5-year overall survival (OS) and cancer-specific survival (CSS).

**Methods:**

We included 4119 patients who were registered from 2004 to 2015 in the Surveillance, Epidemiology, and End Results database. The median follow-up time was 5.8 years. Independent risk factors affecting OS and CSS were identified with univariate and multivariate Cox regression analyses and used to construct nomograms. Internal and external validation were carried out by using the bootstrap method to calculate the concordance indices (C-indices) and plot the calibration curves.

**Results:**

Age, primary site, histological type, T-stage, and treatment were independent risk factors for OS and CSS; marital status and sequence number were factors only for OS. The C-indices for internal validation of OS and CSS were 0.713 (95% CI, 0.697–0.729) and 0.708 (95% CI, 0.688–0.728), respectively, and for external validation they were 0.729 (95% CI, 0.705–0.753) and 0.731 (95% CI, 0.700–0.762), respectively. The calibration curves also revealed good agreement between the predicted and actual survival rates.

**Conclusions:**

We constructed nomograms to predict the 3- and 5-year OS and CSS of patients with uveal melanoma without metastases. Our nomograms may improve prognostication and assist with the development of individualized treatment strategies.

## 1. Introduction

Uveal melanoma (UM) is a common primary intraocular malignancy in adults, mainly originating from pigment and nevus cells within the uveal tissue. UM can be divided into choroidal, ciliary-body, and iris melanoma, with choroidal melanoma accounting for 86.3% of UM cases [1]. The reported age-adjusted incidence of UM is 5.1 per million population in the United States [2]. The onset is typically between the ages of 50 and 70 years, and the clinical manifestations are painless vision loss, visual distortion, and visual field loss, although 30% of patients have no ocular symptoms at the time of diagnosis [3]. The pathogenesis of UM has not been fully elucidated, but well-defined risk factors include a light iris [4], fair skin [5], cutaneous nevi, iris nevi [6], dysplastic nevus syndrome [7], and BRCA1-associated protein-1 (BAP1) tumor predisposition syndrome [8, 9].

UM is highly malignant and prone to metastasis, with the liver being the most common site of metastasis [10]. Due to the lack of lymphatic vessels in the eye, UM mainly metastasizes through the blood. In some patients, UM has already metastasized before clinical diagnosis, and once metastasis occurs, patients have a survival period of only about six months [11]. The current treatment options for UM include laser photocoagulation, photodynamic therapy, transpupillary thermotherapy, radiation, surgical local excision, enucleation, and various combination treatments. Radiation therapies for UM include plaque brachytherapy (BT), stereotactic radiosurgery (SRS), and proton-beam radiotherapy (PBRT) [12].

Currently, the prognosis of uveal melanoma can be predicted by several independent factors without the use of an ideal prognostic prediction model. Moreover, we are aware of no reports on prognostic factors and survival rates of patients with UM without metastases. Nomograms are used as simple statistical tools for the accurate prediction of patient prognoses, with visualized results, with the aim of improving patient outcomes. The Surveillance, Epidemiology, and End Results (SEER) database is the most authoritative source of information on cancer incidence and survival in the United States, covering approximately 28% of the population in 18 different population groups [13]. In this study, we collected data from the SEER database, specifically of patients with UM without metastases registered on the database from 2004 to 2015. Our aim was to create nomograms that accurately predict their overall survival (OS) and cancer-specific survival (CSS), by integrating and visualizing the independent risk factors. In this way, we wanted to improve the assessment of patient prognoses and provide a clinical basis for the development of individualized treatment strategies.

## 2. Materials and Methods

### 2.1. Patient Selection

**Figure 1:**
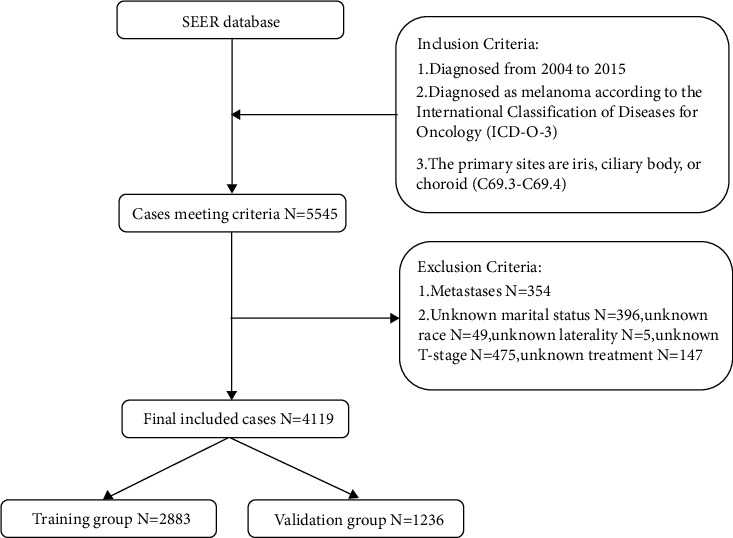
Flow chart of case selection process.

The data were extracted by using the National Cancer Institute SEER*∗*Stat software (seer.cancer.gov/seerstat) version 8.3.9. The site codes for the choroid (C69.3) and the ciliary body and iris (C69.4), as well as several International Classification of Diseases for Oncology codes (ICD-O-3: 8720, 8721, 8722, 8723, 8730, 8740, 8744, 8745, 8761, 8770, 8771, 8772, 8773, and 8774) were used to identify patients with UM. We collected data from 5545 patients who were diagnosed with UM from 2004 to 2015 in the SEER database. We excluded 354 patients with metastases. Further exclusion criteria were as follows: unknown marital status, unknown race, unknown laterality, unknown T-stage, and unknown treatment. Finally, 4119 patients were included in the study, of which we allocated 2883 to the training group and 1236 to the validation group, in a ratio of 7 : 3 (Figure 1). The training group is used for model fitting, while the validation group is used to evaluate the performance of this model. The SEER database is freely available to the public and is updated annually, which is why the ethics committee waived approval and the need for informed consent.

### 2.2. Variables

We recorded patients' age, sex, race, marital status, and year of diagnosis, as well as UM laterality, primary site, histological type, T-stage, treatment, sequence number, and survival time. T-stage classification was performed according to the 6th edition of the American Joint Committee on Cancer (AJCC) staging system, as the 7th edition was only published in 2010. The primary endpoints were OS and CSS. OS was defined as the time from diagnosis to death or the end of follow-up and CSS as the time from diagnosis to UM-related death or the end of follow-up.

### 2.3. Statistical Analysis

The optimal cut-off values for age (57 and 79 years) were determined by using X-tile software, thus dividing the patients into three age groups: ≤57 years, 58–79 years, and ≥80 years. We performed statistical analysis by using IBM SPSS Statistics for Windows version 26.0 (IBM Corp., Armonk, NY, USA). Group allocation was performed by using the random case selection method in a ratio of 7 : 3 for training : validation. We described the numbers and percentages of cases for each baseline characteristic in the overall, training, and validation groups and compared the statistical differences between the training and validation groups by using the chi-square test. The hazard ratios (HRs) and 95% confidence interval (CI) were calculated by using univariate and multivariate Cox regression analyses. The potential risk factors in the training group were determined by using a univariate Cox regression model, and the variables with *P* < 0.05 were included in the multivariate Cox regression analysis for determination of the independent risk factors affecting the OS and CSS.

We used R software 4.0.5 (R Foundation for Statistical Computing, Vienna, Austria) to incorporate the independent risk factors from the multivariate Cox regression analyses into nomograms for the prediction of the 3- and 5-year OS and CSS values for patients with UM without metastases. Internal and external validation of the constructed nomograms were carried out by using the bootstrap method to calculate the concordance indices (C-indices) and plot the calibration curves. The C-index can be used to evaluate the predictive ability of the model. A value of 0.5 indicates that the model is not predictive, values from 0.51 to 0.70 indicate low predictive accuracy, values from 0.71 to 0.90 indicate moderate predictive accuracy, and values above 0.90 indicate high predictive accuracy. All *P*-values in this study were two-tailed, and differences were considered statistically significant when *P* < 0.05.

## 3. Results

### 3.1. Baseline Characteristics

We enrolled a total of 4119 patients with UM without metastases, including 2883 in the training group and 1236 in the validation group, with a median follow-up time of 5.8 years. The differences between the training and validation groups were not statistically significant (*P* > 0.05) (Table 1). Regarding overall patients, the mean age was 62 years (range, 5 to 99 years). The percentages of male and female patients were 52.6% and 47.4%, respectively. The vast majority of patients were white (97.8%). Married, single, divorced or separated, and widowed patients accounted for 66.4%, 14.8%, 8.8%, and 10.0% of patients, respectively. The vast majority of patients had monocular onset (99.9%) of UM, including the left eye of 49.6% of patients and the right eye of 50.3%. The primary site of UM for the vast majority of patients was the choroid (88.7%). The histological types were known in 22.3% of patients, of which 46.5%, 12.9%, and 36.8% were spindle-cell, epithelioid-cell, and mixed epithelioid- and spindle-cell melanoma, respectively; the other 3.7% of cases were rare histological types such as balloon-cell and necrotic-type melanoma. Tumor staging was performed according to the AJCC staging system, 6th edition, with T1, T2, T3, and T4 tumors accounting for 36.8%, 43.6%, 17.2%, and 2.4% of tumors, respectively. A majority of patients were treated with radiation only (69.2%), 22.8% of patients were treated with surgery only, and 8.0% of patients were treated with combined surgery and radiation. The majority of cancers were single primary cancers (71.9%) while a few were multiple primary cancers (28.1%). The other baseline characteristics are summarized in Table 1.

### 3.2. Univariate and Multivariate Cox Regression Analysis

Among the 4119 patients with UM without distant metastases, 1563 patients died during the study period, of which 920 died because of UM, yielding 3- and 5-year OS rates of 83.3% and 73.0%, respectively, and 3- and 5-year CSS rates of 88.5% and 81.8%, respectively. All the possible prognostic factors were included into the univariate Cox regression analysis of the training group (Table 2). As a result, six factors (age, marital status, primary site, histological type, T-stage, and treatment) were associated with both OS and CSS (*P* < 0.05). In addition, sequence number was associated with OS (*P* < 0.05). To exclude the effect of confounding factors, the significant factors were included in a multivariate Cox regression analysis (Table 3). As a result, all seven factors were independently associated with OS (*P* < 0.05), and five factors (age, primary site, histological type, T-stage, and treatment) were associated with CSS (*P* < 0.05).

### 3.3. Construction and Verification of Nomograms

**Figure 2:**
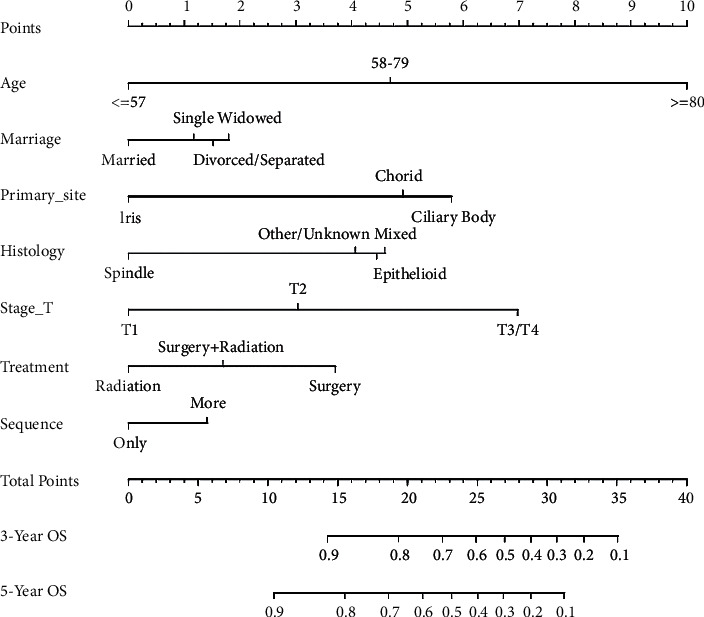
Nomograms for the prediction of the 3- and 5-year OS values for patients with UM without metastases. OS: overall survival.

**Figure 3:**
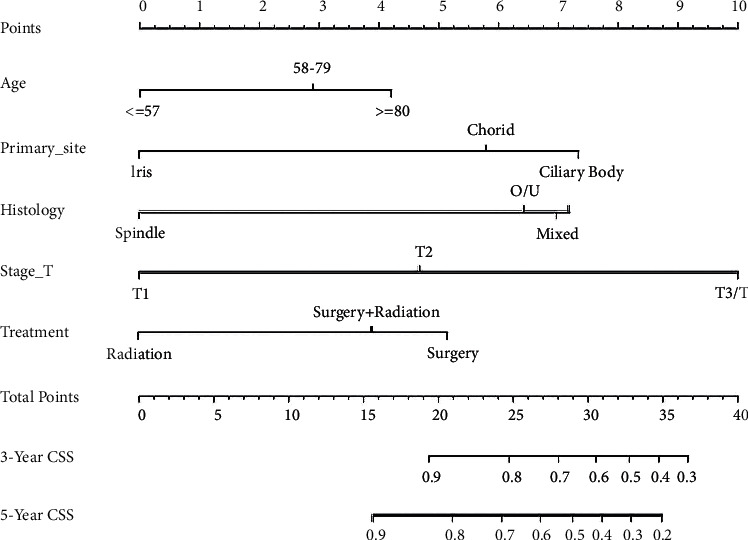
Nomograms for the prediction of the 3- and 5-year CSS values for patients with UM without metastases. CSS: cancer-specific survival.

The constructed nomograms (Figures 2 and 3) are used as follows: a vertical line is made from the point on the axis of each prognostic factor that corresponds to the patient's value to the points axis at the top of the nomogram; the intersection of the line and the points axis is the score assigned to that factor. The points of all prognostic factors are summed to obtain the total points for a patient. Finally, a vertical line is made from the total points on the total-point axis to the 3- and 5-year OS and CSS axes to obtain the survival rates for a patient with specific values for the prognostic factors. We will illustrate its use with a hypothetical patient. The patient is 65 years old, is married, has a single primary choroidal melanoma, and underwent only surgical treatment. The tumor is an epithelioid-cell melanoma, was classified as stage T2, and has not metastasized. According to the nomograms, the total OS score is 21.2 and the total CSS score is 25.9; hence, the 3- and 5-year OS values are approximately 74% and 60%, respectively, while the 3- and 5-year CSS values are approximately 77% and 63%, respectively.

**Figure 4:**
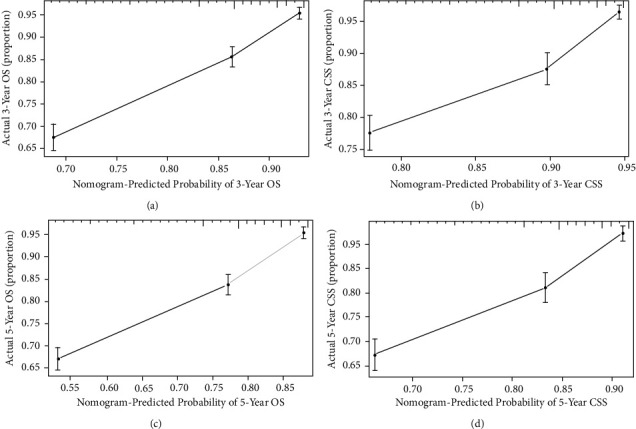
Calibration curves of internal validation for predicting 3-year OS (a), 3-year CSS (b), 5-year OS (c), and 5-year CSS (d). OS: overall survival and CSS: cancer-specific survival.

**Figure 5:**
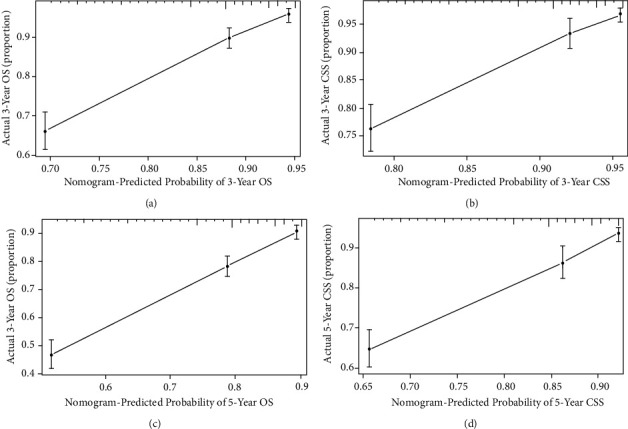
Calibration curves of external validation for predicting 3-year OS (a), 3-year CSS (b), 5-year OS (c), and 5-year CSS (d). OS: overall survival and CSS: cancer-specific survival.

With the bootstrap method in this study, we performed 1000 resamples and obtained C-indices of 0.713 (95% CI, 0.697–0.729) and 0.708 (95% CI, 0.688–0.728), respectively, for the nomograms used to predict OS and CSS during internal validation, and 0.729 (95% CI, 0.705–0.753) and 0.731 (95% CI, 0.700–0.762), respectively, during external validation. We plotted the calibration curves of the internal and external validations (Figures 4 and 5), which revealed good agreement between the predicted and actual survival rates.

## 4. Discussion

Melanoma is a highly malignant cancer of melanocytic origin that can arise from multiple primary sites, such as the skin, mucosa (nose, oropharynx, lungs, gastrointestinal tract, urinary tract, etc.), and eyes (uvea, conjunctiva, cornea, eyelids, orbit, lacrimal gland, etc.). About 5% of melanomas originate in the eye, and of those, about 85% originate in the uvea [14]. Up to 50% of patients with UM develop distant metastases, and once metastases occur, the one-year survival is only 15% [15]. For patients with UM without distant metastases detected at an early stage, the most important issues are prognosis, risk of distant metastases, and survival rate, and these patients expect to choose an individualized treatment plan based on the prognosis. However, to the best of our knowledge, research on the survival and prognostic factors of patients with UM without metastases is sparse. Nomograms are graphs of quantitative analyses that can represent the functional relationship between different variables by a series of parallel line segments in planar coordinates. Nomograms can integrate different prognostic factors to generate specific probabilities of clinical events. Because this tool helps achieve individualized medical treatment, it is widely used in oncology for the development of prognostic models.

In this study, 4119 patients with UM without metastases who were registered in the SEER database were included. After univariate and multivariate Cox regression analyses, we constructed nomograms for the prediction of patients' 3- and 5-year OS and CSS. The evaluation and validation of nomograms may be internal or external. Internal validation is the validation based on the data in the training group, which is part of the model construction; its main purpose is to verify the repeatability of the prediction model and prevent the overfitting of the model. External validation is the application of the constructed prediction model to the validation group; this process includes the calculation of the predicted values for comparison with the actual values to assess whether the prediction results of the prediction model are reliable. Internal and external validation revealed good agreement between the predicted and actual survival rates, indicating that these nomograms are accurate tools for the prediction of survival rates.

UM is most often observed in patients of advanced age. We demonstrated that higher age was associated with lower survival rates. This may be due to the fact that younger patients have a higher proportion of iris melanoma, which is more likely to be far from the central macula and optic disc, with smaller tumor diameter and thickness, and a lower chance of tumor spread compared to other types of UM, while older patients have poorer underlying conditions and more complications [16]. The univariate analyses in this study revealed that sex was not a risk factor for a worse prognosis; however, the *P*-value was exactly 0.05, which was the threshold value selected in this study. The impact of sex on the survival of patients with UM is controversial. Some studies have demonstrated that sex is not associated with the prognosis of patients with UM [17], while it has also been reported that men have a worse prognosis with a higher risk of metastasis than women, which may be related to sex hormones [18]. Marital status is an important factor affecting people's psychological status. Previous studies have shown that marital status has a statistically significant impact on the prognosis of various cancers (lung, colorectal, breast, prostate, ovarian, and pancreatic) [19]. In this study, the best prognosis was observed in married patients and the worst in widowed patients, which is consistent with the results of previous studies [20]. Patients typically receive support and encouragement from their spouses, which helps them better tolerate treatment and stay healthy for a longer time than widowed patients [21]. The financial support of a spouse also increases the patient's chances to obtain suitable treatment and medication support, further increasing their confidence in overcoming the disease [22].

The vast majority of patients with UM have monocular disease; in this study, there were only three cases of bilateral disease. We observed no statistically significant difference in the survival rates between monocular and bilateral disease, which is consistent with the findings of previous studies [23]. As for the primary site, we discovered that iris melanoma yielded the best prognosis, probably because of its smaller size, which facilitates early diagnosis and treatment. In contrast, ciliary-body melanoma had a poor prognosis. First, its tumor site is hidden and difficult to diagnose. Second, the ciliary body is rich in blood vessels and the contraction of the ciliary muscle increases the chance of transvascular metastasis. Finally, the proportion of epithelioid cells in ciliary-body melanoma is high [24]. In a study of 3,432 cases of UM, choroidal and ciliary-body melanoma had a mortality rate at least 10 times higher than iris melanoma [25]. Another study revealed a statistically significant positive correlation between the degree of ciliary-body involvement and the odds of tumor metastasis: 100% involvement of the ciliary body was 3.6 times more likely to result in metastasis than choroidal melanoma [26]. Spindle-cell melanoma was the most common histological type in this study and had the best prognosis, while the epithelioid- and mixed-cell types had a poor prognosis. This may be because of the fact that spindle cells are adhesive cells with intermediate junctions and filamentous cell protrusions, which have a strong cohesive force, lowering the probability of metastasis. In contrast, epithelioid cells are nonadhesive cells with high mobility and poor cohesion; hence, they can easily enter the vascular lumen through the spaces between the basement membrane and endothelial cells, leading to hematogenous metastasis [27].

Tumor stage is an important marker of prognosis, as there were few patients with stage T4 UM without metastasis; we combined patients with stage T3 and those with stage T4 tumors into one group for analysis. We observed that the survival rate gradually decreased with an increase in T-stage. Previous studies demonstrate that the average age of diagnosis gradually increases as T-stage increases, as does the tumor thickness, tumor diameter, and the risks of subretinal fluid, Bruch's membrane rupture, and extraocular spread [28].

The main treatment options for UM are surgery and radiation. Surgical treatment includes enucleation and local excision. Enucleation is the traditional method, although it is no longer the first choice for treating UM because the distant metastasis of the tumor may be accelerated by the pulling and squeezing of the vascular tissue during surgery [29]. As local excision yields fewer complications and can be used to preserve partial vision, it is one of the more desirable methods of treating UM. However, residual tumor tissue and local recurrence are high after local resection, and postoperative supplemental radiation can reduce recurrence and metastasis. Radiation treatment for UM includes plaque BT and teletherapy. The former is a local radiation treatment in which the radiation source is placed on the scleral surface in the same plane as the tumor; the latter includes SRS and PBRT. SRS is used to lyse and kill tumor cells by damaging their DNA, RNA, proteins, chromosomes, and biofilm systems. PBRT is a method of irradiating tumors with the use of a cyclotron to generate high-energy charged particles that converge into a particle beam, which causes tumor cell necrosis and/or vascular occlusion. In our study, patients treated with radiotherapy alone had the best prognosis, while those treated with surgery alone had a poorer prognosis. One study, in which 3291 patients were analyzed by using a propensity score-matching method, demonstrated better survival with radiation than with surgery, especially for patients with early T-stage UM [30]. Another study demonstrated no statistically significant differences in the survival rates yielded by brachytherapy and distant radiotherapy [31]. Taken together, the choice of the treatment method should take various factors into account, such as tumor size, primary site, histological type, and the patient's general condition.

“Multiple primary cancers” refers to the simultaneous or sequential occurrence of two or more independent primary malignancies. In our study, the majority of patients had single primary cancers, and they had a better OS than patients with multiple primary cancers. We concluded that the sequence number did not affect the CSS, as most patients with multiple primary cancers died because of other secondary tumors or systemic diseases. Previous studies have shown that patients with UM have a 9% increased risk of developing a second malignancy, such as cutaneous melanoma, leukemia, thyroid cancer, kidney tumors, and tumors of other parts of the eye or orbit, compared to healthy individuals. On the other hand, there was an 8% increased risk of developing UM after the first diagnosis of any of these other tumors [32].

The present study is subject to certain limitations. First, the results would be more convincing if the prediction model were validated in a multicenter study with large-scale data from other databases or research centers. We are conducting research in our own hospital to verify the findings of this article, and the results will be presented in our next article. Second, some important prognostic indicators are not included in the SEER database, such as tumor thickness, mitotic activity, degree of lymphocyte infiltration, karyotype, and gene expression profile. Finally, there were missing data for some patients regarding the specific histological type. However, if we removed these patients from analyses, the number of cases would be reduced substantially, lowering the accuracy of the results. Despite these limitations, we believe that the prognostic nomograms are useful for the prediction of individual survival rates for patients with UM without metastases.

## 5. Conclusions

Advanced age, ciliary-body involvement, epithelioid-cell melanoma, more advanced T-stage, and surgery-only treatment are independent risk factors for both a poor OS and a poor CSS in patients with UM without metastasis. Widowhood and multiple primary cancers are independent risk factors only for OS. We constructed nomograms to predict the 3- and 5-year OS and CSS, providing a potentially more accurate and individualized method to predict patient survival. These nomograms may ultimately serve as a basis for the development of individualized treatment strategies.

## Figures and Tables

**Table 1:** Baseline characteristics of training group and validation group.

	Overall (*N* = 4119)		Training group (*N* = 2883)		Validation group (*N* = 1236)		*P* value
	Number	Percent	Number	Percent	Number	Percent	
Age, years							0.750
≤57	1550	37.6	1078	37.4	472	38.2	
58–79	2111	51.3	1478	51.3	633	51.2	
≥80	458	11.1	327	11.3	131	10.6	
Sex							0.315
Male	2167	52.6	1502	52.1	665	53.8	
Female	1952	47.4	1381	47.9	571	46.2	
Race							
White	4030	97.8	2819	97.8	1211	98.0	0.884
Black	27	0.7	20	0.7	7	0.6	
Other	62	1.5	44	1.5	18	1.5	
Marital status							0.773
Married	2733	66.4	1900	65.9	833	67.4	
Single	611	14.8	430	14.9	181	14.6	
Divorced/separated	363	8.8	257	8.9	106	8.6	
Widowed	412	10.0	296	10.3	116	9.4	
Year of diagnosis							0.674
2004–2007	1358	33.0	953	33.1	405	32.8	
2008–2011	1224	29.7	886	30.0	358	29.0	
2012–2015	1537	37.3	1064	36.9	473	38.3	
Laterality							0.450
Left	2044	49.6	1422	49.3	622	50.3	
Right	2072	50.3	1458	50.6	614	49.7	
Bilateral	3	0.1	3	0.1	0	0	
Primary site							0.450
Choroid	3652	88.7	2563	88.9	1089	88.1	
Ciliary body	396	9.6	275	9.5	121	9.8	
Iris	71	1.7	45	1.6	26	2.1	
Histological type							0.858
Spindle cell	428	10.4	295	10.2	133	10.8	
Epithelioid cell	119	2.9	85	2.9	34	2.8	
Mixed cell	339	8.2	233	8.2	106	8.6	
Other	34	0.8	26	0.9	8	0.6	
Unknown	3199	77.7	2244	77.8	955	77.3	
T-stage							0.183
T1	1516	36.8	1053	36.5	463	37.5	
T2	1797	43.6	1241	43.0	556	45.0	
T3	709	17.2	516	17.9	193	15.6	
T4	97	2.4	73	2.5	24	1.9	
Treatment							0.660
Surgery only	940	22.8	647	22.4	293	23.7	
Radiation only	2851	69.2	2007	69.6	844	68.3	
Surgery + radiation	328	8.0	229	7.9	99	8.0	
Sequence number							0.608
Only one	2960	71.9	2065	71.6	895	72.4	
More	1159	28.1	818	28.4	341	27.6	

**Table 2:** Univariate Cox regression analysis of OS and CSS in the training group.

	OS		CSS	
	HR (95% CI)	*P* value	HR (95% CI)	*P* value
Age, years		**<0** **.00** **1** ^**∗**^		**<0** **.00** **1** ^**∗**^
≤57	1		1	
58–79	2.128 (1.837–2.465)		1.576 (1.326–1.873)	
≥80	5.236 (4.380–6.260)		2.005 (1.543–2.605)	
Sex		0.050		0.383
Male	1		1	
Female	0.888 (0.789–1.000)		0.934 (0.800–1.089)	
Race		0.379		0.687
White	1		1	
Black	0.607 (0.252–1.461)		0.619 (0.199–1.926)	
Other	0.797 (0.470–1.350)		1.078 (0.594–1.957)	
Marital status		**<0** **.00** **1** ^**∗**^		**0** **.00** **2** ^**∗**^
Married	1		1	
Single	1.058 (0.886–1.263)		0.850 (0.668–1.083)	
Divorced/separated	1.265 (1.032–1.551)		1.260 (0.977–1.625)	
Widowed	2.154 (1.831–2.534)		1.432 (1.129–1.817)	
Year of diagnosis		0.577		0.856
2004–2007	1		1	
2008–2011	1.033 (0.897–1.189)		1.013 (0.842–1.217)	
2012–2015	0.946 (0.805–1.113)		0.956 (0.780–1.172)	
Laterality		0.563		0.144
Left	1		1	
Right	1.052 (0.935–1.184)		1.115 (0.956–1.302)	
Bilateral	1.638 (0.409–6.566)		2.855 (0.711–11.465)	
Primary site		**0** **.01** **1** ^**∗**^		**0** **.00** **8** ^**∗**^
Choroid	1		1	
Ciliary body	1.232 (1.029–1.476)		1.342 (1.067–1.689)	
Iris	0.508 (0.253–1.019)		0.420 (0.157–1.123)	
Histological type		**<0** **.00** **1** ^**∗**^		**<0** **.00** **1** ^**∗**^
Spindle cell	1		1	
Epithelioid cell	2.845 (2.027–3.993)		3.851 (2.464–6.018)	
Mixed cell	2.442 (1.873–3.185)		3.348 (2.327–4.816)	
Other/unknown	1.282 (1.032–1.592)		1.556 (1.139–2.126)	
T-stage		**<0** **.00** **1** ^**∗**^		**<0** **.00** **1** ^**∗**^
T1	1		1	
T2	1.573 (1.356–1.825)		1.867 (1.517–2.299)	
T3/T4	3.273 (2.788–3.843)		4.622 (3.729–5.728)	
Treatment		**<0** **.00** **1** ^**∗**^		**<0** **.00** **1** ^**∗**^
Surgery only	1		1	
Radiation only	0.564 (0.494–0.643)		0.473 (0.399–0.560)	
Surgery + radiation	0.717 (0.573–0.897)		0.817 (0.625–1.067)	
Sequence number		**<0** **.00** **1** ^**∗**^		0.228
Only one	1		1	
More	1.494 (1.321–1.689)		0.897 (0.752–1.070)	

HR: hazard ratio, CI: confidence interval, OS: overall survival, and CSS: cancer-specific survival. ^*∗*^*P* < 0.05.

**Table 3:** Multivariate Cox regression analysis of OS and CSS in the training group.

	OS		CSS	
	HR (95% CI)	*P* value	HR (95% CI)	*P* value
Age, years		**<0** **.00** **1** ^**∗**^		**<0** **.00** **1** ^**∗**^
≤57	1		1	
58–79	2.005 (1.723–2.332)	**<0** **.00** **1** ^**∗**^	1.501 (1.262–1.785)	**<0** **.00** **1** ^**∗**^
≥80	4.384 (3.601–5.339)	**<0** **.00** **1** ^**∗**^	1.804 (1.386–2.348)	**<0** **.00** **1** ^**∗**^
Marital status		**0.004**		0.174
Married	1		1	
Single	1.189 (0.994–1.423)	0.059	0.853 (0.667–1.090)	0.203
Divorced/separated	1.253 (1.020–1.539)	0.032	1.186 (0.918–1.532)	0.195
Widowed	1.304 (1.095–1.553)	**0** **.00** **3** ^**∗**^	1.142 (0.887–1.470)	0.305
Primary site		**0** **.04** **6** ^**∗**^		**0** **.04** **4** ^**∗**^
Choroid	1		1	
Ciliary body	1.135 (0.944–1.366)	0.178	1.244 (0.983–1.573)	0.069
Iris	0.484 (0.240–0.978)	**0** **.04** **3** ^**∗**^	0.437 (0.162–1.175)	0.101
Histological type		**<0** **.00** **1** ^**∗**^		**<0** **.00** **1** ^**∗**^
Spindle cell	1		1	
Epithelioid cell	1.929 (1.367–2.720)	**<0** **.00** **1** ^**∗**^	2.740 (1.746–4.299)	**<0** **.00** **1** ^**∗**^
Mixed cell	1.971 (1.508–2.576)	**<0** **.00** **1** ^**∗**^	2.669 (1.853–3.836)	**<0** **.00** **1** ^**∗**^
Other/unknown	1.823 (1.442–2.304)	**<0** **.00** **1** ^**∗**^	2.472 (1.770–3.451)	**<0** **.00** **1** ^**∗**^
T-stage		**<0** **.00** **1** ^**∗**^		**<0** **.00** **1** ^**∗**^
T1	1		1	
T2	1.567 (1.348–1.822)	**<0** **.00** **1** ^**∗**^	1.936 (1.568–2.390)	**<0** **.00** **1** ^**∗**^
T3/T4	2.804 (2.379–3.306)	**<0** **.00** **1** ^**∗**^	4.096 (3.289–5.100)	**<0** **.00** **1** ^**∗**^
Treatment		**<0** **.00** **1** ^**∗**^		**<0** **.00** **1** ^**∗**^
Surgery only	1		1	
Radiation only	0.579 (0.489–0.685)	**<0** **.00** **1** ^**∗**^	0.484 (0.391–0.598)	**<0** **.00** **1** ^**∗**^
Surgery + radiation	0.743 (0.586–0.943)	**0** **.01** **4** ^**∗**^	0.838 (0.630–1.116)	0.227
Sequence number		**0** **.00** **1** ^**∗**^		
Only one	1			
More	1.232 (1.087–1.396)	**0** **.00** **1** ^**∗**^		

HR: hazard ratio, CI: confidence interval, OS: overall survival, and CSS: cancer-specific survival. ^*∗*^*P* < 0.05.

## Data Availability

The data used to support the findings of this study are available from the first author upon request.
